# HSF1 is involved in suppressing A1 phenotype conversion of astrocytes following spinal cord injury in rats

**DOI:** 10.1186/s12974-021-02271-3

**Published:** 2021-09-16

**Authors:** Lilan Li, Yu Li, Bingqiang He, Hui Li, Huiyuan Ji, Yingjie Wang, Zhenjie Zhu, Yuming Hu, Yue Zhou, Ting Yang, Chunshuai Sun, Ying Yuan, Yongjun Wang

**Affiliations:** 1 Key Laboratory of Neuroregeneration of Jiangsu and Ministry of Education, Co-innovation Center of Neuroregeneration, Nantong University, 19 Qixiu Road, Nantong, 226001 People’s Republic of China; 2 Department of Neurosurgery, Tongji Hospital, Tongji Medical College, Huazhong University of Science and Technology, Wuhan, 430030 People’s Republic of China; 3 Department of Rehabilitation Medicine, Affiliated Hospital of Nantong University, Nantong, 226001 People’s Republic of China

**Keywords:** HSF1, HSP70, Spinal cord, Astrocyte, Inflammation, Phenotype

## Abstract

**Background:**

Two activation states of reactive astrocytes termed A1 and A2 subtypes emerge at the lesion sites following spinal cord injury (SCI). A1 astrocytes are known to be neurotoxic that participate in neuropathogenesis, whereas A2 astrocytes have been assigned the neuroprotective activity. Heat shock transcription factor 1 (HSF1) plays roles in protecting cells from stress-induced apoptosis and in controlling inflammatory activation. It is unknown whether HSF1 is involved in suppressing the conversion of A1 astrocytes following SCI.

**Methods:**

A contusion model of the rat spinal cord was established, and the correlations between HSF1 expression and onset of A1 and A2 astrocytes were assayed by Western blot and immunohistochemistry. 17-AAG, the agonist of HSF1, was employed to treat the primary cultured astrocytes following a challenge by an A1-astrocyte-conditioned medium (ACM) containing 3 ng/ml of IL-1α, 30 ng/ml of TNF-α, and 400 ng/ml of C1q for induction of the A1 subtype. The effects of 17-AAG on the phenotype conversion of astrocytes, as well as underlying signal pathways, were examined by Western blot or immunohistochemistry.

**Results:**

The protein levels of HSF1 were significantly increased at 4 days and 7 days following rat SCI, showing colocalization with astrocytes. Meanwhile, C3-positive A1 astrocytes were observed to accumulate at lesion sites with a peak at 1 day and 4 days. Distinctively, the S100A10-positive A2 subtype reached its peak at 4 days and 7 days. Incubation of the primary astrocytes with ACM markedly induced the conversion of the A1 phenotype, whereas an addition of 17-AAG significantly suppressed such inducible effects without conversion of the A2 subtype. Activation of HSF1 remarkably inhibited the activities of MAPKs and NFκB, which was responsible for the regulation of C3 expression. Administration of 17-AAG at the lesion sites of rats was able to reduce the accumulation of A1 astrocytes.

**Conclusion:**

Collectively, these data reveal a novel mechanism of astrocyte phenotype conversion following SCI, and HSF1 plays key roles in suppressing excessive increase of neurotoxic A1 astrocytes.

**Supplementary Information:**

The online version contains supplementary material available at 10.1186/s12974-021-02271-3.

## Background

Astrocytes are the most abundant cell population in the central nervous system (CNS) that perform a wide array of functions, including neurotransmitter recycling, regulation of synaptogenesis, blood-brain barrier (BBB) formation and maintenance, milieu homeostasis, and immune signaling [1–3]. Once there is trauma or pathogenesis of the CNS, astrocytes are inducibly activated and transferred to the two adaptive states termed A1 and A2 phenotypes by analogy with the M1 and M2 macrophages [4–6]. A1 astrocytes have been found to release neurotoxic factors such as complement components and inflammatory cytokines to mediate the death of neurons and oligodendrocytes, thereby contributing to the progression of neuropathology [7, 8]. Indeed, they are shown to participate in a variety of neurodegenerative diseases, including Alzheimer’s disease, Parkinson’s disease, and multiple sclerosis [6]. As such, A1 reactive astrocytes are defined as a neuroinflammatory phenotype that has harmful functions [2, 4, 6, 7]. Conversely, ischemia-induced A2 astrocytes displayed neuroprotective functions by producing anti-inflammatory cytokines and neurotrophic factors [4, 7, 9, 10]. Thereafter, they have been induced to promote the recovery and repair of CNS [6, 11]. Spinal cord injury (SCI) will result in astrocyte reactivity which impacts on the functional outcomes. Dynamic polarization of reactive astrocytes with at least two states (A1 and A2) emerges across the pathological process of the cord spanning from several days to months [4, 12]. However, the temporal-spatial conversion of the two states of astrocytes and the underlying regulatory mechanisms are not fully elucidated.

Complement component 3 (C3) is exclusively expressed by A1 astrocytes and has been used as a signature to differ from the S100A10-specific A2 astrocytes [2, 4]. Investigation from murine models has shown that cytokines TNF-α, IL-1α, and C1q secreted by microglia exposed to LPS are efficient in promoting an A1 astrocyte phenotype [4]. These studies have provided new clues to shed light on activation states or heterogeneity of astrocytes, as well as their potential involvements in pathology following SCI. It is known that injury-induced A1 astrocytes at lesion sites of the cord are increasingly replaced by A2 astrocytes with the progression of neuropathology, and several signal pathways are found to control the transition of cell states [2, 5, 10, 13, 14]. For example, blocking of the Notch signal pathway is able to suppress the activation of neurotoxic A1 astrocytes after spinal cord injury, due to affecting STAT3 activity [14], as JAK/STAT3, together with MAPK and NF-κB pathway, is an important initiator and modulator of astrocyte reactivity [15–18]. The importance of STAT3 in mediating the conversion of astrocyte phenotypes has already been proven in the AD model of mice, in which the deficiency of STAT3 is able to promote A1 astrocytes switching to A2 states [19]. Nevertheless, the key regulator(s) involved in state control of astrocyte reactivity following SCI remains unclear.

Heat shock transcription factor 1 (HSF1) is a member of a family of DNA-binding proteins that mediates transcription of heat shock proteins (HSPs) for the proper folding, trafficking, and degradation of misfolded proteins in response to diverse stresses [20, 21]. The biological importance of HSF1 is recognized by its contribution to protecting various cells against stress-induced apoptosis [22–25], inhibiting inflammatory cytokine expression in macrophages [26–28] and mediating neurogenesis and extraembryonic development [29–31]. In unstressed cells, the inactive HSF1 monomer is bound in the cytoplasm and forms a complex with heat shock proteins (HSPs) 40, 70, and 90, as well as the cytosolic chaperonin TCP1 ring complex (TRiC) [20]. However, tissue injury or environmental stresses such as elevated temperature and oxidative stress will lead to the activation of HSF1 by releasing from the complex and translocation to the nucleus, where it binds a heat shock response element (HSE) to induce transcription of downstream genes [23, 26, 32]. The neuropathological process following SCI is associated with cellular stresses by affecting activation of inflammation, reactive gliosis, and neuronal survival [33]. Though HSF1 has been shown to be dynamically regulated in the injured cord [34], its exact roles in the regulation of cell events have not been fully unveiled. Given that endogenous HSF1 is sufficient in inhibiting the expression of inflammatory cytokine TNF-α and IL-1β, the hallmarks of A1 astrocyte secretion [4, 27, 28], it is assumed that HSF1 may act to control reactive astrocyte states following SCI. In the present study, the relations between temporal-spatial expression of HSF1 and states of reactive astrocytes were analyzed following rat SCI. The conversion of astrocytes was also observed in the presence of HSF1 agonist in vivo and in vitro. Accordingly, the signal pathways implicated in the activation of reactive astrocytes were also examined. Our results displayed that HSF1 was efficient in suppressing the A1 phenotype conversion of astrocytes following rat SCI.

## Methods

### Animals

Adult male Sprague–Dawley (SD) rats, weighing 180–220 g, were provided by the Center of Experimental Animals, Nantong University. All animal care, breeding, and testing procedures were approved according to the Animal Care and Use Committee of Nantong University and the Jiangsu Province Animal Care Ethics Committee. All animals were housed in individual cages in a temperature and light/dark cycle controlled environment with free access to food and water.

### Establishment of contusion SCI rat model

The number of animals subjected to surgical treatment was calculated by six per experimental group in triplicate. The contusion SCI rat model was prepared as previously described [35]. Briefly, rats were anesthetized with an intraperitoneal injection of 10% chloral hydrate (3 mg/kg). The fur was shaved from the surgical site and the skin was disinfected with chlorhexidine. A 15-mm midline skin incision was made to expose the vertebral column. After the spinal thoracic region was exposed by separation of the dorsal muscles to the side, the spinous processes of the T8–T10 vertebrae were exposed. A laminectomy was performed at the vertebral level T9, exposing the dorsal cord surface with the dura remaining intact. The exposed spinal cord segment (about 3 mm in length) received a 150-kilodyne spinal contusion injury using the IH-0400 Impactor (Precision Systems and Instrumentation) injury device. The impact rod was removed immediately, and the wound was irrigated. Muscles and incisions were sutured using silk threads. Postoperative care included butorphanol administration twice a day for a 5-day period, as well as vitamins, saline, and enrofloxacin twice a day for a 7-day period. Manual expression of bladders was performed twice a day until animals recovered spontaneous voiding.

### Cell culture and treatment

Astrocytes were prepared from the spinal cord of newborn Sprague–Dawley rats, 1–2 days after birth, and the astrocytes were isolated and cultured according to previously described methods [36]. Briefly, the cells were enzymatically dissociated using 0.25% trypsin (Gibco-BRL) for 6 min at 37 °C, and the suspension was then centrifuged at 1200 rpm for 5 min and cultured in 1:1 Dulbecco’s modified Eagle’s medium: Ham’s F-12 medium supplemented with 10% fetal bovine serum (FBS), 0.224% NaHCO_3_, and 1% penicillin/streptomycin in the presence of 5% CO_2_. A monolayer of astrocytes was obtained 12–14 days after the plating. Non-astrocytes were detached from the flasks by shaking and were removed by changing the medium. Third- or fourth-passage cells were rendered quiescent through incubation in a medium containing 0.5% FBS for 4 days prior to the experiments. Astrocyte phenotypes were confirmed by cells exhibiting a characteristic morphology and positive staining for the astrocytic marker glial fibrillary acid protein (GFAP).

The primary astrocytes were treated with heat shock for 1 h at 42 °C, followed by a recovery for 0 h or 3 h at 37 °C. For evaluation of the effects of HSF1 intervention on the astrocyte phenotype, the cells were treated with 0–10 μM 17-allylamino-17-demethoxygeldanamycin (17-AAG, Sigma) or with 0–60 μM quercetin (NEW ENGLAND BioLabs) dissolved in 0.1% DMSO for 24 h. Alternatively, the primary astrocytes were transfected with HSF1 siRNA (sense strand 5'-GCC UAU UGA GGC AGA GAA UdTdT-3′, antisense strand 5′-A UUC UCU GCC UCA AUA GGC dTdT-3′) or scramble siRNA (sense strand 5′-GGC UCU AGA AAA GCC UAU GC dTdT-3′, antisense strand 5′-GC AUA GGC UUU UCU AGA GCC dTdT-3′) with iMAX transfection reagent (Invitrogen) for 48 h. Immediately after treatment, cells were subjected to Western blot analysis.

### Induction of the A1 phenotype of astrocytes

Generation of A1 astrocytes referred to the method of Liddelow et al. [4]. Briefly, astrocytes were cultured in DMEM medium in the presence of IL-1α (3 ng/ml, Peprotech, 400-01), TNF-α (30 ng/ml, CST, 8902), and C1q (400 ng/ml, Novus protein, NBP2-62410) (named Astrocyte-Conditional-Medium, ACM) for 24 h, and the conversion of states was examined by C3 expression.

### Western blot

Protein was extracted from cells with a buffer containing 1% SDS, 100 mM Tris-HCl, 1 mM PMSF, and 0.1 mM β-mercaptoethanol, following treatment with heat shock or 0-10 μM 17-AAG. Alternatively, protein was extracted from 1-cm spinal segments of the injured site at 0 day, 1 day, 4 days, and 1 week following contusion (*n* = 6 in each sample). The protein concentration of each specimen was detected by the Bradford method to maintain the same loads. Protein extracts from tissues were treated with 400 units of lambda protein phosphatase (NEB) and 1 mM MnCl_2_ for 1 min before being heat denatured at 95 °C for 5 min. The samples were electrophoretically separated on 10% SDS-PAGE and transferred to PVDF membranes. The membranes were subjected to the reaction with a 1:1000 dilution of primary antibodies in TBS buffer at 4 °C overnight, followed by a reaction with secondary antibody conjugated with goat anti-rabbit or goat anti-mouse HRP dilution 1:1000 (Santa Cruz) at room temperature for 2 h. After the membrane was washed, the HRP activity was detected using an ECL kit. The image was scanned with a GS800 Densitometer Scanner (Bio-Rad), and the data were analyzed using the PDQuest 7.2.0 software (Bio-Rad). β-actin (1:5000) was used as an internal control. Antibodies used in Western blot are rabbit anti-C3 antibody (Abcam); rabbit anti-S100A10 antibody (Abcam); rabbit anti-HSF1 antibody (CST, 12972); rabbit anti-HSP70 antibody (Life Sciences); p65NFκB, p-ERK1/2, ERK1/2, JNK, p-JNK, p-P38, P38 (cell signaling technology; CST); and β-actin (Proteintech).

### Subcellular fractionation

Isolation of cytosolic and nuclear fraction was referred to the method of Ahn and Thiele [22]. Briefly, the cells were washed in PBS, followed by lysis in 400 μl of buffer containing 10 mM HEPES at pH 7.9, 10 mM KCl, 0.1 mM EDTA, 0.1 mM EGTA, 1 mM DTT, 5 mg/ml leupeptin, and 0.5 mM PMSF on ice for 5 min. NP40 at a final concentration of 0.6% was then added, and the mixture was centrifuged at 16,000 g at 4 °C for 1 min. The supernatant (cytosolic fraction) was collected, whereas the pellet (nuclear fraction) was resuspended in buffer containing 20 mM HEPES at pH 7.9, 0.4 M NaCl, 10 mM KCl, 1 mM EDTA, 1 mM EGTA, 1 mM DTT, 0.01% Triton X-100, and 0.5 mM PMSF on ice for 15 min, and centrifuged at 4 °C, 16,000 g for 15 min, and the supernatant was collected as a nuclear extract. Proteins of nuclear and cytosolic extracts were detected by Western blot. Both GAPDH and H3 were used as an internal control of cytoplasmic and nuclear proteins, respectively.

### Tissue immunohistochemistry

The vertebra segments were harvested from 6 experimental models of each time point, post-fixed and sectioned. Sections were allowed to incubate with rabbit anti-C3 antibody (1:200 dilution, Abcam), rabbit anti-S100A10 antibody (1:200 dilution, Abcam), rabbit anti-HSF1 antibody (1:200 dilution, CST, 12972), mouse anti-S100β antibody (1:400 dilution, Sigma), or mouse anti-human GFAP antibody (1:400 dilution, Sigma) at 4 °C for 36 h. The sections were further reacted with the FITC-labeled secondary antibody goat anti-mouse IgG (1:400 dilution, Gibco), or the TRITC-labeled secondary antibody donkey anti-rabbit IgG (1:400 dilution, Gibco) at 4 °C overnight, followed by observation under a confocal laser scanning microscope (Leica, Heidelberg, Germany).

### Quantitative analysis of GFAP-positive area at lesion sites of the spinal cord

The percentage of GFAP-positive area at lesion site of the spinal cord before or after 17-AAG treatment was analyzed by NIH ImageJ software as described by Fedorova and Pavel [37]. Briefly, a lesion border with 2000 μm rostral and caudal to the epicenter of the GFAP-positive sections was selected and suffered to image cropping by Adobe Photoshop software. Digital RGB images of the spinal cord were converted to 8-bit grayscale (Image > Color > Split Channels) by ImageJ. After RGB color separation, the color thresholds were set with Image > Adjust > Threshold, so that the GFAP immunostaining could be colored red. Area statistics (the area, area fraction expressed as %) were set up (Analyze > Set Measurements) prior to analysis, then measurements were taken based on the existing area selection in the thresholded binary image (Analyze > Measure), and finally, the results were automatically recorded and displayed in the results panel.

### Statistical analysis

Statistical analysis used the GraphPad Prism 8 software (San Diego, CA, USA). All data were presented as means ± SD. Comparisons between two groups following normal distribution were analyzed by a two-tailed unpaired Student’s *t* test or the Mann-Whitney test when the distribution was not parametric. Differences between multiple groups were analyzed using one­way or two-way analysis of variance (ANOVA), followed by Dunnett’s or Tukey’s post hoc test. A *P* value < 0.05 was considered statistically significant and was denoted in the figures as *P* < 0.05.

## Results

### SCI-induced activation of HSF1 is correlated to the conversion of A1 and A2 astrocyte phenotypes

**Fig. 1:**
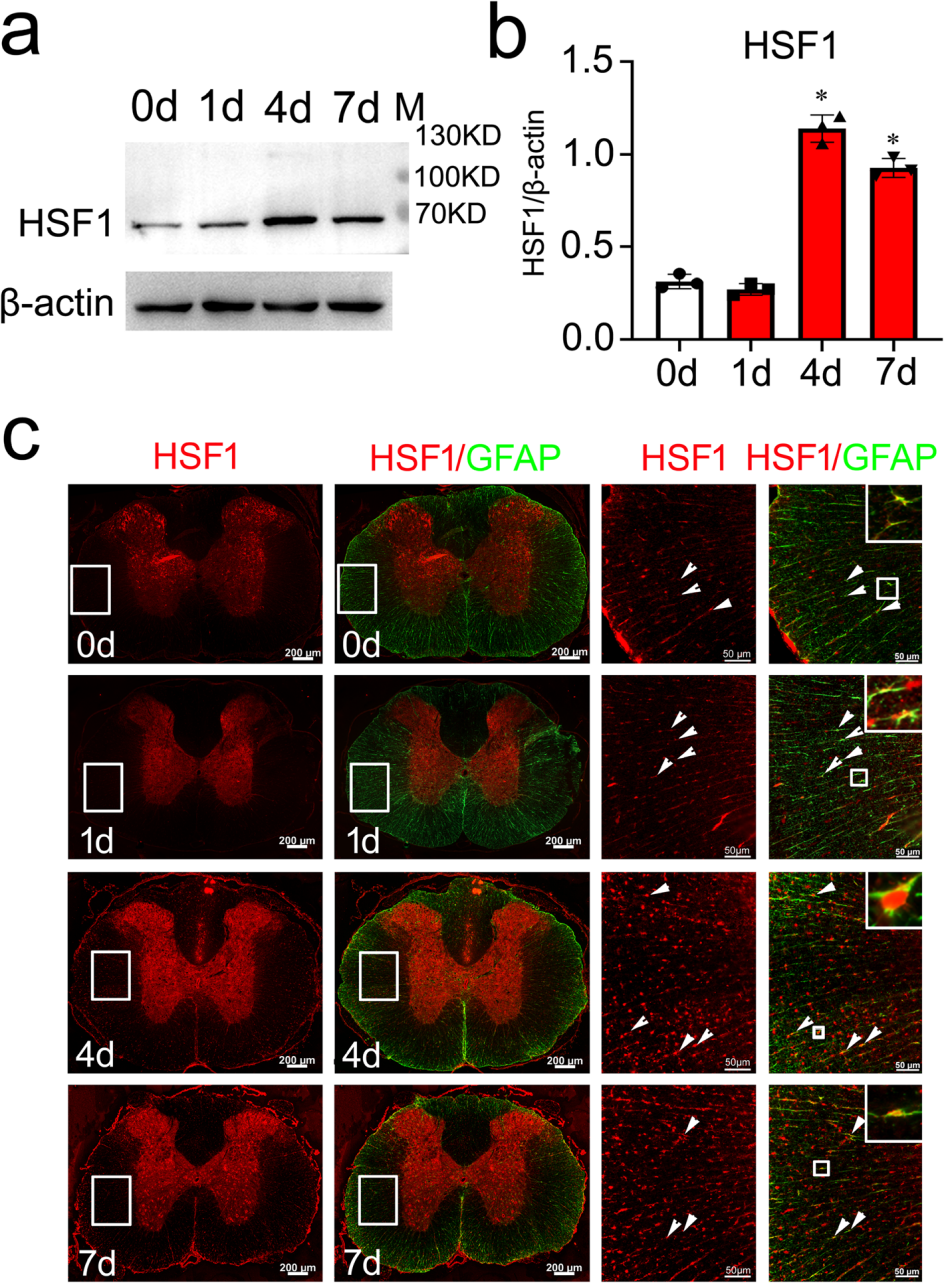
Expression analysis of HSF1 protein levels and colocalization with astrocytes following SCI. **a** Western blot analysis of HSF1 expression following spinal cord contusion at 0 day, 1 day, 4 days, and 7 days, respectively. M indicates the markers. **b** Quantification data as shown in (**a**); quantities were normalized to endogenous β-actin. *n* = 6. Experiments were performed in triplicates. Error bars represent the standard deviation. *P < 0.05, one-way ANOVA with Dunnett’s post hoc test. *F* (3, 8) = 61.05, *P* < 0.0001. 4 days vs 0 day, *P* < 0.0001; 7 days vs 0 day, *P* = 0.0002. **c** Immunostaining of HSF1 in the cross sections of rat contused spinal cord showed colocalization with GFAP-positive cells at 0 day, 1 day , 4 days, and 7 days, respectively. The rectangles indicate region magnified. Arrowheads indicate colocalization of HSF1 with astrocytes. Scale bars, 200 μm or 50 μm in magnification

To understand the correlations between HSF1 activation and conversion of reactive astrocyte states following SCI, injury-induced expression of HSF1 in the contused spinal cord was first determined. Results displayed that protein levels of HSF1 were significantly increased at 4 days and 7 days at lesion sites of the cord, while it was unchanged at 1 day (Fig. 1a, b). The expression of HSF1 in the sham group was simultaneously examined and showed no changes (Additional file 1). Further immunohistochemistry was carried out to observe the distribution of HSF1 in the astrocytes. Cord sections were made from a 0.25-cm length to the epicenter of the contusion. Results demonstrated that HSF1 was detected to colocalize with GFAP- and S100β-positive astrocytes at different time points following SCI (Fig. 1c; Additional file 2), indicating potential regulatory roles of the protein on the cell events of astrocytes.

**Fig. 2:**
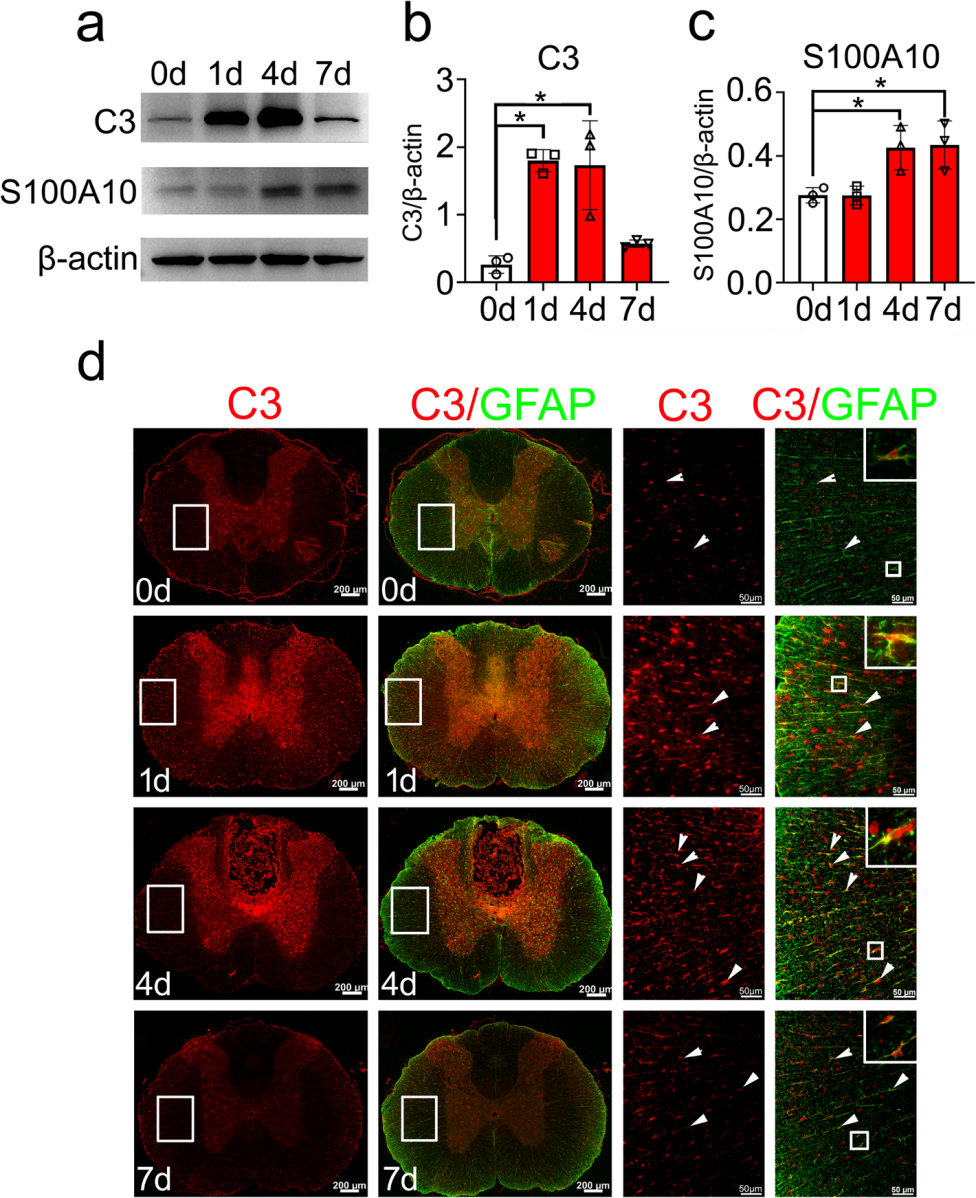
Examination of C3-positive A1 astrocytes following SCI. **a–c** Western blot analysis of C3 (**a, b**) and S100A10 (**a, c**) protein levels following spinal cord contusion at 0 day, 1 day, 4 days, and 7 days, respectively. Quantities were normalized to endogenous β-actin. *n* = 6. Experiments were performed in triplicates. Error bars represent the standard deviation, *P < 0.05, one-way ANOVA with Dunnett’s post hoc test. For C3 quantitative analysis, *F* (3, 8) = 15.66, *P* = 0.0010. 1 day vs 0 day, *P* = 0.0018; 4 day vs 0 day, *P* = 0.0024. For S100A10 quantitative analysis, *F* (3, 8) = 7.930, *P* = 0.0088. 4 day vs 0 day, *P* = 0.0305; 7 days vs 0 day, *P* = 0.0233. **d** Examination of C3-positive A1 astrocytes in the cross sections of rat contused spinal cord at 0 day, 1 day, 4 days, and 7 days, respectively. Rectangles indicate the region magnified. Arrowheads indicate C3-positive astrocytes. Scale bars, 200 μm or 50 μm in magnification

**Fig. 3:**
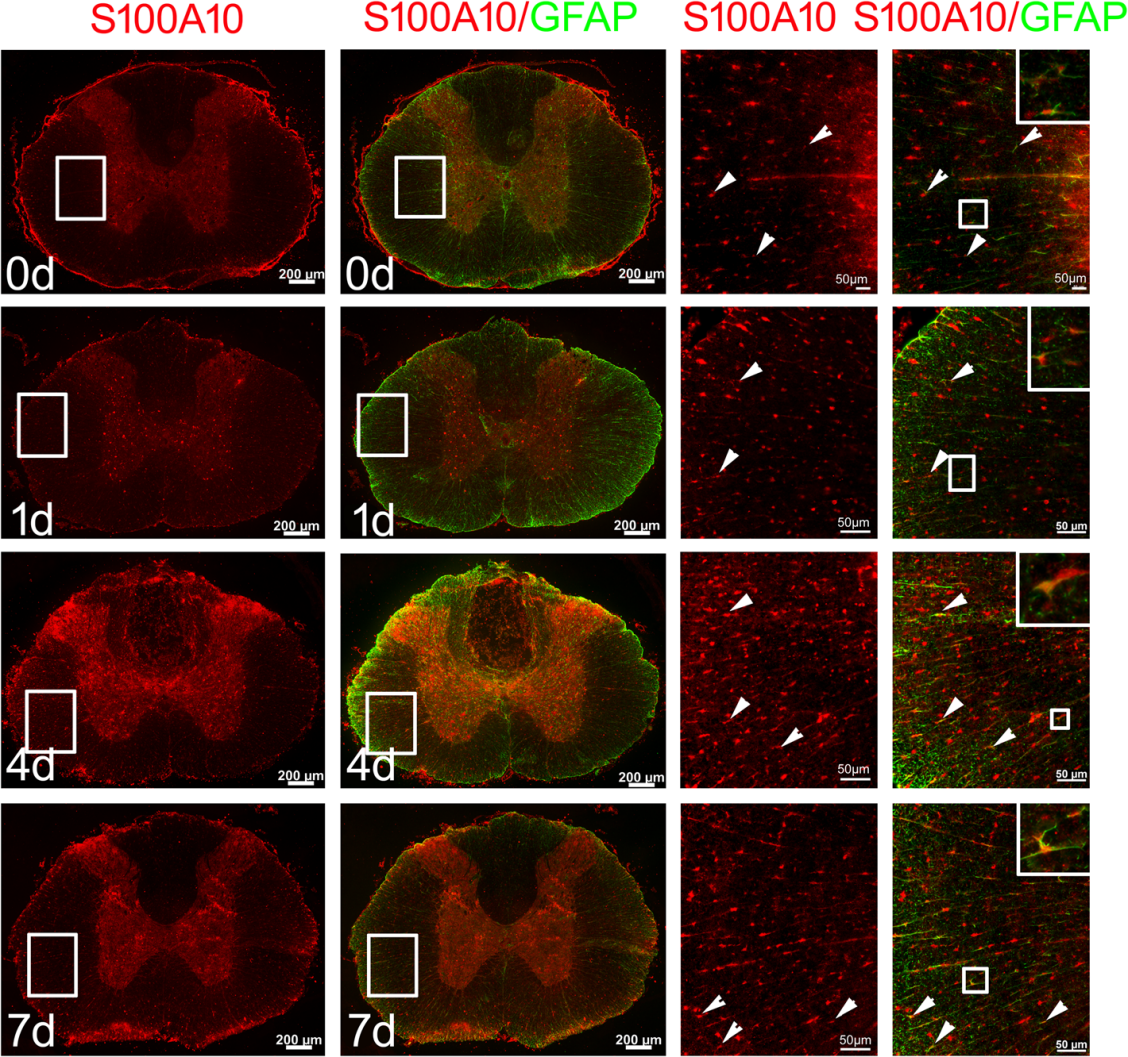
Examination of S100A10-positive A2 astrocytes in the cross sections of rat contused spinal cord at 0 days, 1 days, 4 days, and 7 days, respectively. Rectangles indicate the region magnified. Arrowheads indicate S100A10-positive astrocytes. Scale bars, 200 μm or 50 μm in magnification

Next, we examined the expression of complement component C3 and S100A10 protein, the specific marker of A1 and A2 reactive astrocytes, respectively [4], in parallel with those of HSF1 in the injured cord. Western blots demonstrated that the protein level of C3 was rapidly induced at 1 day and 4 days, whereas it was significantly decreased at 7 days after cord contusion. Comparatively, expression of S100A10 was markedly enhanced at 4 days and 7 days (Fig. 2a–c). Immunostaining demonstrated that most reactive astrocytes were C3-positive at 1 day and 4 days (Fig. 2d), while S100A10-positive astrocytes primarily emerged at 4 days and 7 days in the injured cord (Fig. 3). These data indicate that SCI-induced activation of HSF1 is potentially involved in the phenotype conversion of A1 and A2 astrocytes.

### HSF1 responds to heat shock stress in astrocytes

**Fig. 4:**
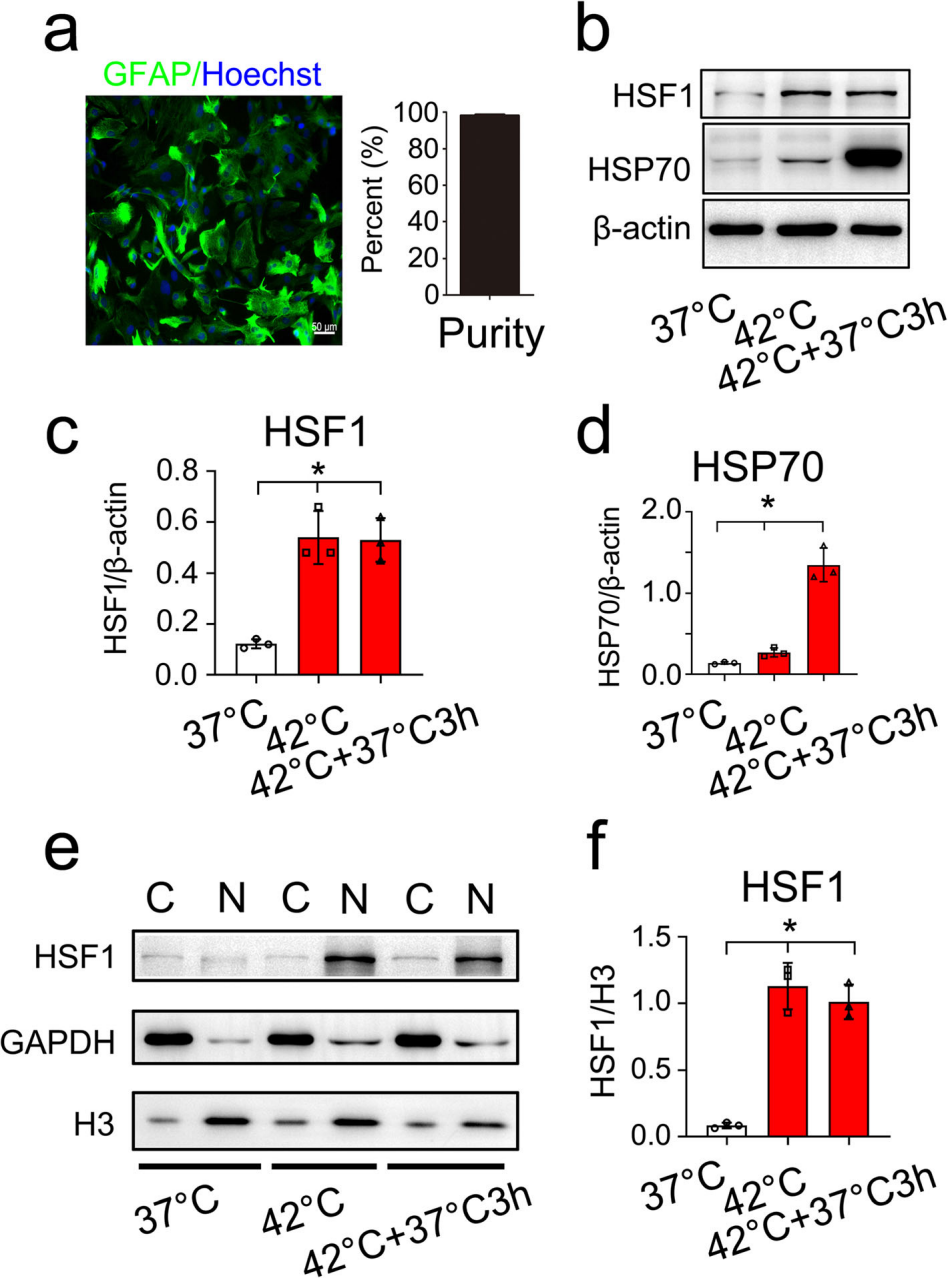
Heat shock stress induces activation of HSF1 in the astrocytes. **a** Showing purified primary astrocytes stained with GFAP and Hoechst 33342; **b–d** Examination of HSF1 (**b**, **c**) and HSP70 (**b**, **d**) protein levels in the astrocytes treated with heat shock for 1 h at 42 °C, followed by a recovery for 0 h or 3 h at 37 °C. Quantities were normalized to endogenous β-actin. Experiments were performed in triplicates. Error bars represent the standard deviation. *P < 0.05, one-way ANOVA with Dunnett’s post hoc test. For HSF1 quantitative analysis, *F* (2, 6) = 27.72, *P* = 0.0009. 42 °C vs 37 °C, *P* = 0.0011; 42 °C + 37 °C 3 h vs 37 °C, *P* = 0.0013. For HSP70 quantitative analysis, *F* (2, 6) = 87.75, *P* = 0.0098. 42 °C vs 37 °C, *P* = 0.0019; 42°C+ 37°C 3 h vs 37°C, *P* = 0.0144. **e**, **f** Immunoblotting of HSF1 in the cytoplasm and nucleus of astrocytes following heat shock stress. Quantities were normalized to endogenous GAPDH (cytoplasm) or histone H3 (nucleus). Experiments were performed in triplicates. Error bars represent the standard deviation. *P < 0.05, one-way ANOVA with Dunnett’s post hoc test. *F* (2, 6) = 61.92, *P* < 0.0001. 42 °C vs 37 °C, *P* = 0.0001, 42 °C + 37 °C 3 h vs 37 °C, *P* = 0.0002. Scale bars, 50 μm

It is still unclear whether the heat shock responses exist in the astrocytes of rats, which might participate in neuropathology in the context of various stresses. To unveil the potential protective mechanism in the stressed astrocytes, the rat astrocytes were firstly isolated and cultured with a purity of more than 95% (Fig. 4a). The astrocytes were then subjected to heat shock for 1 h at 42°C, followed by a recovery for 0 h or 3 h at 37 °C. HSF1 subcellular distribution was assessed by biochemical fractionation and immunoblotting experiments before and after exposure to heat shock. As shown in Fig 4b–d, the protein levels of HSF1 and the downstream HSP70 were significantly increased by the heat stress. HSF1 was found predominantly in the nucleus, rather than in the cytosolic fraction in response to heat shock (Fig. 4e, f). The data indicate that HSF1 in astrocytes is responsive to environmental stresses such as heat shock.

### Activation of HSF1 is able to inhibit expression of C3 in the astrocytes

**Fig. 5:**
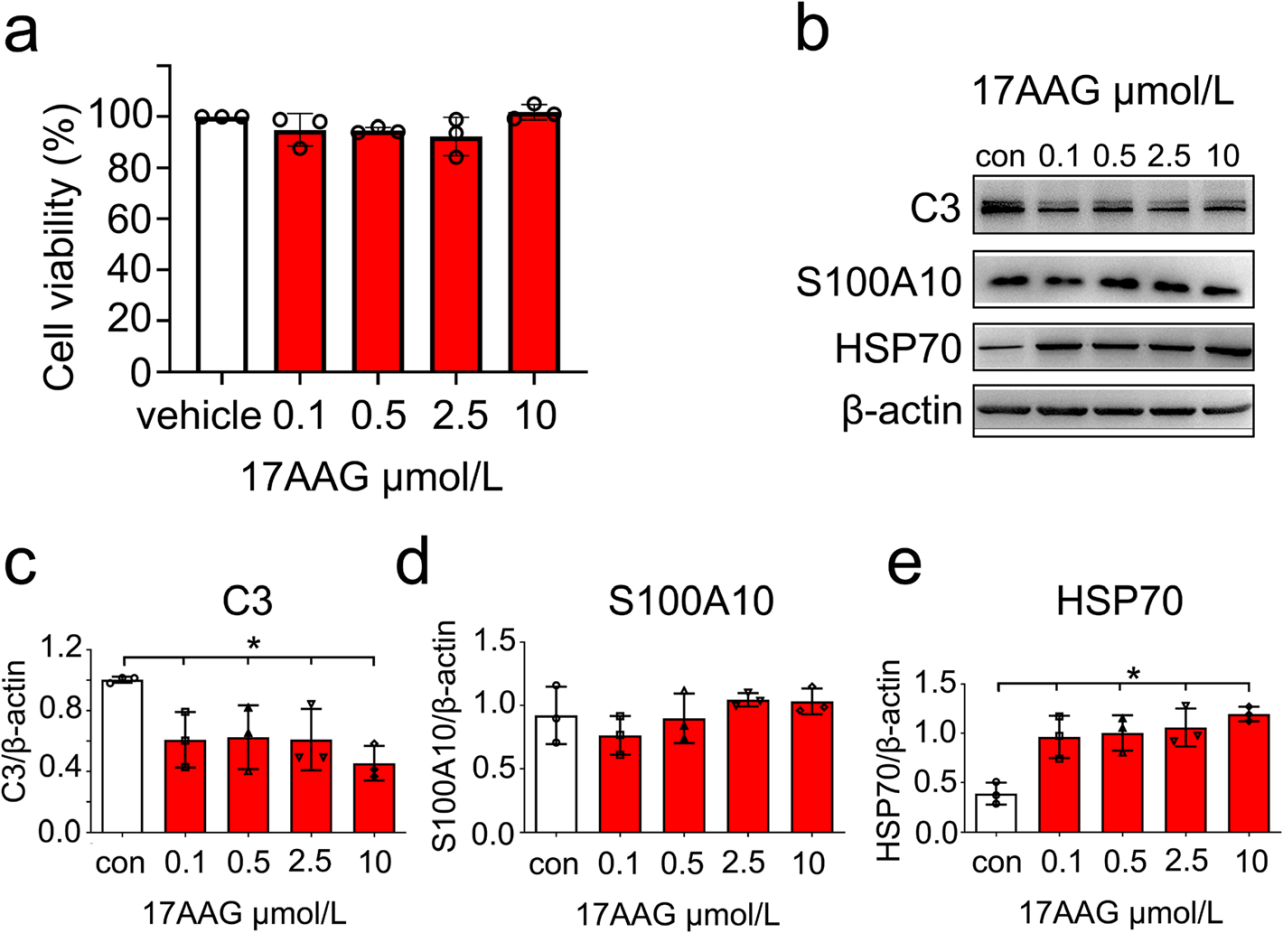
Effects of HSF1 agonist 17-AAG on the expression of C3 and S100A10 in the astrocytes. **a** Toxic assay of 0-10 μmol/L 17-AAG for the astrocytes. **b–e** Western blot analysis of C3 (**b**, **c**), S100A10 (**b**, **d**) and HSP70 (**b**, **e**) in astrocytes following cell incubation at 0–10 μmol/L 17-AAG for 24 h. Quantities were normalized to endogenous β-actin. Experiments were performed in triplicates. Error bars represent the standard deviation, *P < 0.05, one-way ANOVA with Dunnett’s post hoc test. For C3 quantitative analysis, *F* (4, 10) = 4.763, *P* = 0.0207. 0.1 vs con, *P* = 0.0019; 0.5 vs con, *P* = 0.0526; 2.5 vs con, *P* = 0.0432; 10 vs con, *P* = 0.0066. For HSP70 quantitative analysis, *F* (4, 10) = 10.89, *P* = 0.0011. 0.1 vs con, *P* = 0.0051; 0.5 vs con, *P* = 0.0032; 2.5 vs con, *P* = 0.0017; 10 vs con, *P* = 0.0004

To ascertain the function of HSF1 in suppressing the conversion of A1 reactive astrocytes, primary astrocytes were stimulated with 0–10 μM 17-AAG, the agonist of HSF1 for 24 h. The agonist was shown non-toxic to the astrocytes (Fig. 5a), but it was able to attenuate C3 protein levels of the cells without affecting the expression of S100A10 (Fig. 5b, d). Upregulation of HSP70 by the addition of 17-AAG validated that HSF1 was efficiently activated in the glial cells (Fig. 5e). The data indicate that activation of HSF1 is able to inhibit C3 expression of astrocytes.

**Fig. 6:**
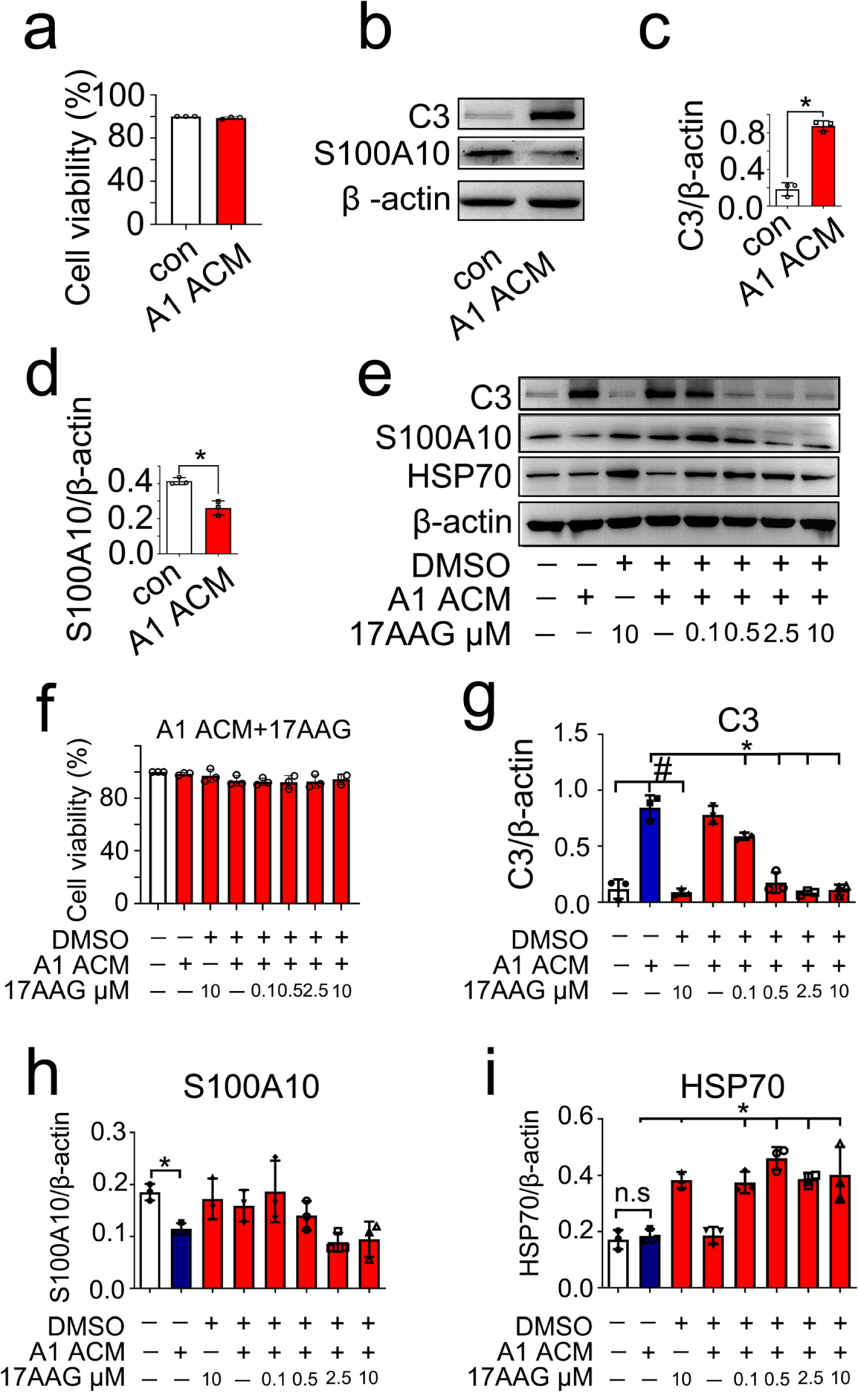
Effects of HSF1 agonist 17-AAG on the conversion of astrocyte phenotypes. **a** Toxic assay of A1-astrocyte-conditioned medium (ACM) containing 3 ng/ml IL-1α, 30 ng/ml TNF-α, and 400 ng/ml C1q in the DMEM for the astrocytes. **b–d** Effects of ACM on the conversion of astrocytes evaluated by C3 (**b**, **c**) and S100A10 (**b**, **d**) protein levels following cell incubation for 24 h. Quantities were normalized to endogenous β-actin. Experiments were performed in triplicates. Error bars represent the standard deviation, *P < 0.05. The data were analyzed by two­tailed unpaired Student’s *t* test. *P* = 0.0002 in (**c**) and *P* = 0.0042 in (**d**). **e–i** Determination of C3 (**e**, **g**), S100A10 (**e**, **h**), and HSP70 (**e**, **i**) in astrocytes following cell incubation at ACM for 24 h in the presence of 0-10 μmol/L 17-AAG. **f** Toxic assay of ACM in the presence of 0–10 μmol/L 17-AAG for the astrocytes. Quantities were normalized to endogenous β-actin. Experiments were performed in triplicates. Error bars represent the standard deviation, *P < 0.05, ^#^*P* < 0.05, one-way ANOVA with Tukey’s post hoc test. For C3 quantitative analysis in (**g**), F (7, 16) = 66.95, *P* = 0.0207. ACM vs con, *P* < 0.0001; 10 μmol/L 17-AAG + DMSO vs ACM, *P* < 0.0001; 0.1 μmol/L 17-AAG + ACM + DMSO vs ACM, *P* = 0.0103; 0.5 μmol/L 17-AAG + ACM + DMSO vs ACM, *P* < 0.0001; 2.5 μmol/L 17-AAG + ACM + DMSO vs ACM, *P* < 0.0001; 10 μmol/L 17-AAG + ACM + DMSO vs ACM, *P* < 0.0001. For S100A10 quantitative analysis in (**h**), *F* (7, 16) = 4.324, *P* = 0.0073. ACM vs con, *P* = 0.018. For HSP70 quantitative analysis in (**i**), *F* (7, 16) = 19.79, *P* < 0.0001. ACM vs con, *P* = 0.8082; 10 μmol/L 17-AAG + DMSO vs ACM, *P* = 0.0019; 0.1 μmol/L 17-AAG + ACM + DMSO vs ACM, *P* = 0.0029; 0.5 μmol/L 17-AAG + ACM + DMSO vs ACM, *P* < 0.0001; 2.5 μmol/L 17-AAG + ACM + DMSO vs ACM, *P* = 0.0015; 10 μmol/L 17-AAG + ACM + DMSO vs ACM, *P* < 0.0001

To elucidate the roles of HSF1 in suppressing the conversion of A1 astrocytes, the A1 astrocytes were induced by A1-astrocyte-conditioned medium (ACM) containing 3 ng/ml IL-1α, 30 ng/ml TNF-α, and 400 ng/ml C1q in the DMEM for 24 h [4]. Assay of CCK-8 revealed that the ACM in the presence or absence of 17-AAG did not affect the cell viability of astrocytes (Fig. 6a, f). However, induction of the ACM efficiently promoted conversion of the astrocytes to the A1 phenotype, as shown by an increase of C3 and a reduction of S100A10 protein levels (Fig. 6b–d). An addition of 0–10 μM 17-AAG to the ACM demonstrated that the expression of C3 in astrocytes was significantly attenuated by activation of HSF1, as shown by enhancement of its chaperons HSP70. However, the protein levels of A2 astrocyte-specific S100A10 remained unchanged (Fig. 6e–i). The data indicate that activation of HSF1 is able to suppress the expression of C3 in the reactive astrocytes.

To gain an insight into the effects of HSF1 loss of function on the astrocyte phenotype, the cells were transfected with HSF1 siRNA for 48 h or treated with 0–60 μM quercetin, an inhibitor of HSF1 protein for 24 h [38]. Unexpectedly, knockdown or inhibition of HSF1 expression resulted in a decrease in C3 protein levels, in either the presence or absence of A1 ACM (Additional file 3). The data indicate that HSF1 deficiency also has a negative effect on A1 phenotype conversion of astrocytes.

### HSF1 inhibits expression of C3 through modulation of mitogen-activated protein kinases

**Fig. 7:**
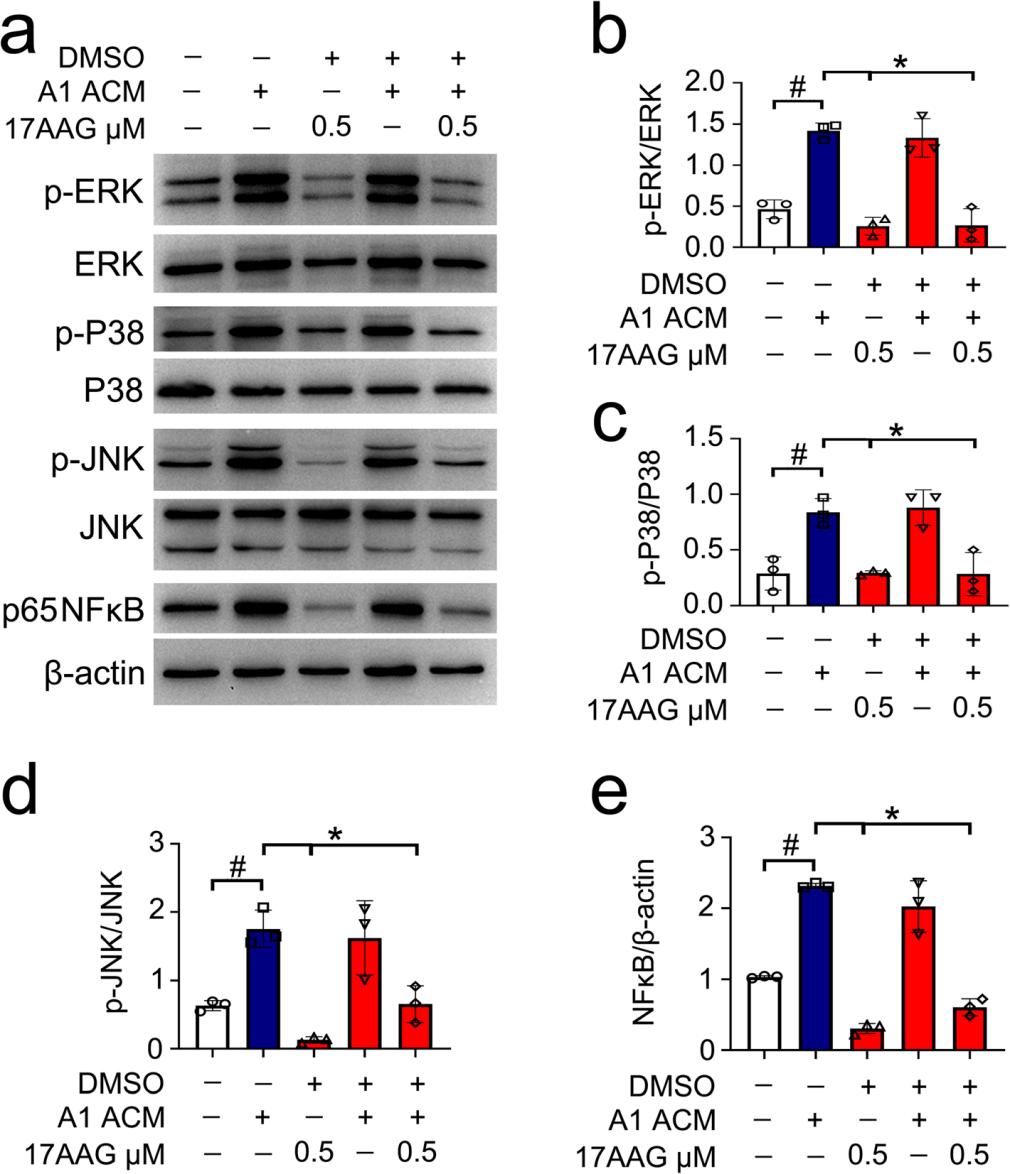
Western blot analysis of HSF1 agonist 17-AAG on the activation of ERK (**a**, **b**), P38 (**a**, **c**), JNK (**a**, **d**), and NFκB (**a**, **e**) following cell incubation at ACM for 24 h in the presence of 0.5 μmol/L 17-AAG. Quantities were normalized to endogenous β-actin. Experiments were performed in triplicates. Error bars represent the standard deviation, *P < 0.05, ^#^*P* < 0.05, one-way ANOVA with Tukey’s post hoc test. For quantitative analysis of ERK activation, *F* (4, 10) = 39.91, *P* < 0.001. ACM vs con, *P* = 0.0003; 17-AAG + DMSO vs ACM, *P* < 0.0001; 17-AAG + ACM + DMSO vs ACM, *P* < 0.0001. For quantitative analysis of P38 activation, *F* (4, 10) = 14.52, *P* = 0.0004. ACM vs con, *P* = 0.0056; 17-AAG + DMSO vs ACM, *P* = 0.0053; 17-AAG + ACM + DMSO vs ACM, *P* = 0.0059. For quantitative analysis of JNK activation, *F* (4, 10) = 16.46, *P* = 0.0002. ACM vs con, *P* = 0.0097; 17-AAG + DMSO vs ACM, *P* = 0.0006; 17-AAG + ACM + DMSO vs ACM, *P* = 0.0112. For quantitative analysis of NFκB activation, *F* (4, 10) = 76.38, *P* < 0.0001. ACM vs con, *P* < 0.0001; 17-AAG + DMSO vs ACM, *P* < 0.0001; 17-AAG + ACM + DMSO vs ACM, *P* < 0.0001

The expression of C3 in astrocytes has been shown under control of MAPKs in response to inflammatory stimuli [39]. To shed light on the mechanism of HSF1 inhibiting the conversion of A1 astrocytes, phosphorylation of ERK1/2, JNK, and P38 was determined following astrocyte incubation at A1 ACM for 24h with or without addition of 0.5 μM 17-AAG. Results displayed that activation of HSF1 with 17-AAG remarkably decreased the phosphorylation of ERK1/2, JNK, and P38 proteins. Also, the expression of p65NFκb was accordingly reduced (Fig. 7a–e). The data indicate that forced expression of HSF1 results in significant suppression of MAPK phosphorylation during conversion of A1 astrocytes.

### Administration of HSF1 agonist to the contused spinal cord of rat decreases the number of A1 astrocytes

**Fig. 8:**
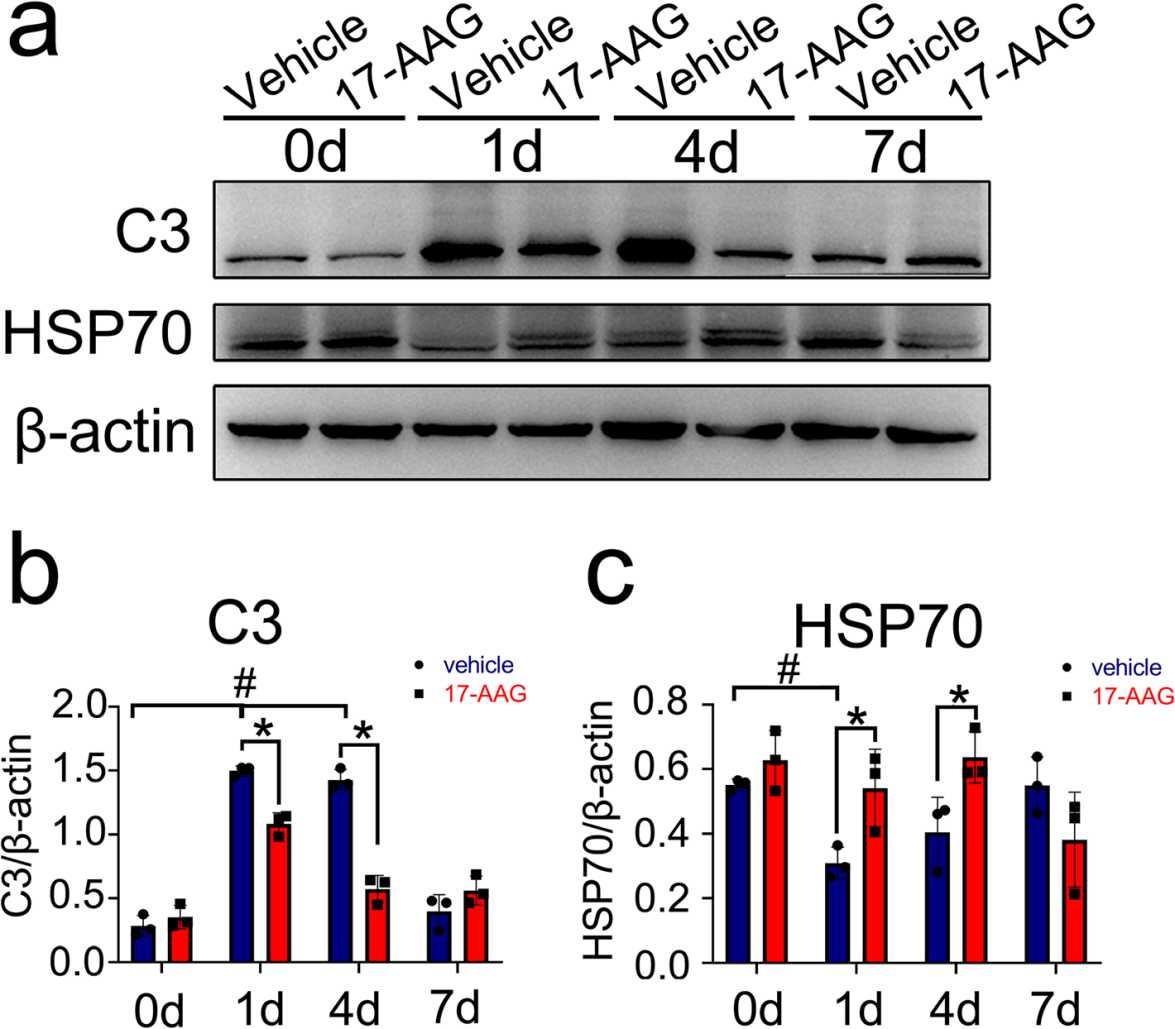
Western blot analysis of C3 and HSP70 protein levels in the injured cord. The rats were received 100 μl intraperitoneal injections of 20 mg/kg 17-AAG three times a week on alternate days. **a–c** Western blot analysis of C3 (**a**, **b**) and HSP70 (**a**, **c**) protein levels at 0 day, 1 day, 4 days, and 7 days following spinal cord injury. Quantities were normalized to endogenous β-actin. *n* = 6. Experiments were performed in triplicates. Error bars represent the standard deviation, *P < 0.05, ^#^*P* < 0.05, two-way ANOVA with Tukey’s test. For C3 quantitative analysis, *F* (3, 6) = 78.80, *P* < 0.0001. 1 day vehicle vs 0 day, *P* < 0.0001; 4 days vehicle vs 0 day, *P* < 0.0001; 1 day 17-AAG vs 1 day vehicle, *P* = 0.0028; 4 days 17-AAG vs 4 days vehicle, *P* < 0.0001. For HSP70 quantitative analysis, *F* (3, 6) = 6.709, *P* = 0.0241. 1 day vehicle vs 0 day, *P* = 0.0244; 1 day 17-AAG vs 1 day vehicle, *P* = 0.0279; 4 days 17-AAG vs 4 days vehicle, *P* = 0.0419

**Fig. 9:**
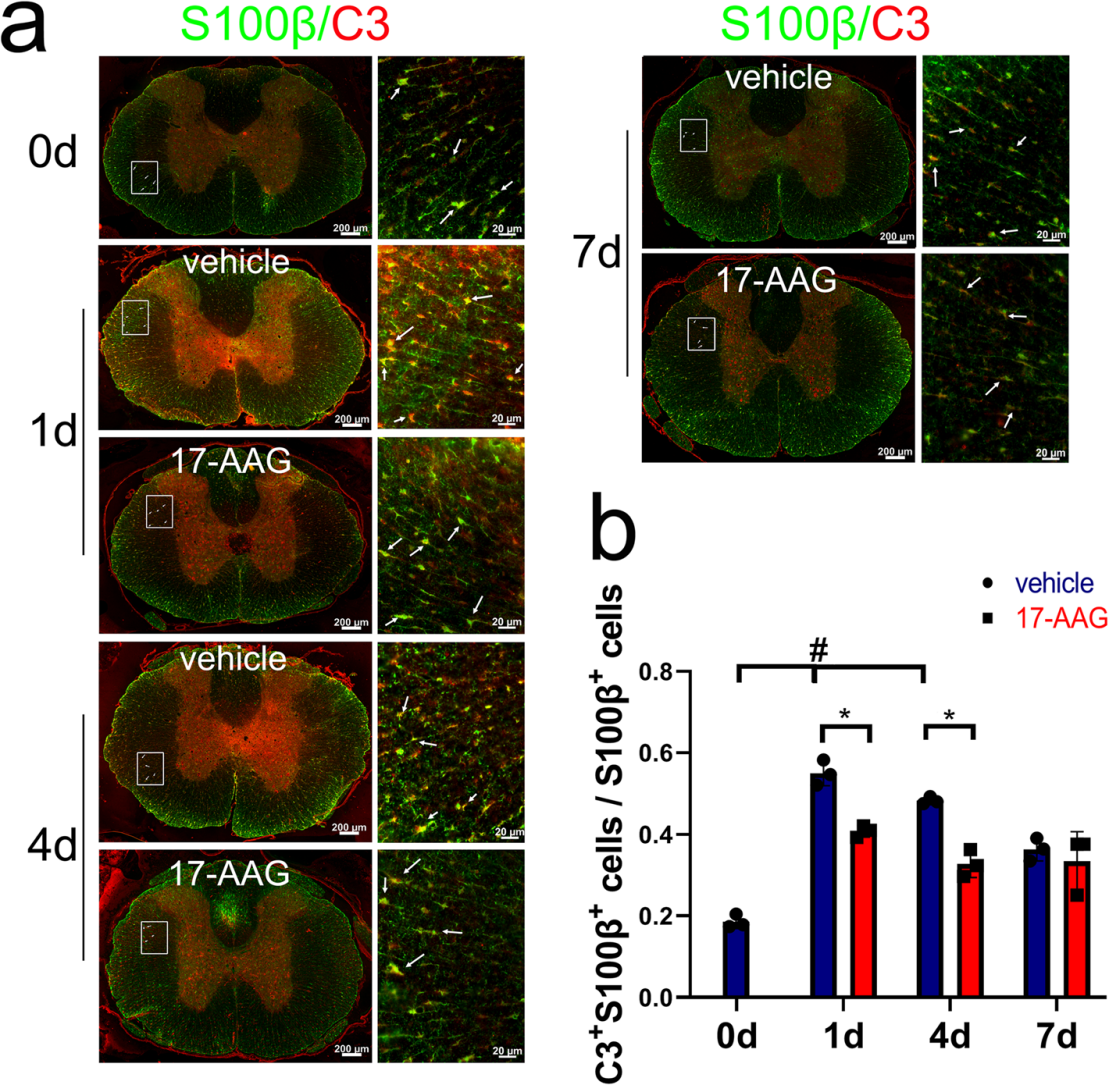
Immunostaining of C3-positive astrocytes at lesion sites of the injured cord following treatment with 17-AAG. The rats were received 100 μl intraperitoneal injections of 20 mg/kg 17-AAG three times a week on alternate days. **a** Immunostaining of C3-positive astrocytes at lesion sites treated with 17-AAG for 0 day, 1 day, 4 days, and 7 days, respectively. **b** Statistical analysis of (**a**) in triplicates each 50 fields. Error bars represent the standard deviation, *P < 0.05, ^#^*P* < 0.05, two-way ANOVA with Tukey’s test. F (3, 6) = 12.89, *P* = 0.0050. 1 day vehicle vs 0 day, *P* < 0.001; 4 days vehicle vs 0 day, *P* = 0.0001; 1 day 17-AAG vs 1 day vehicle, *P* = 0.0072; 4 days 17-AAG vs 4 days vehicle. *P* = 0.0046. Rectangles indicate the region magnified. Arrows indicate C3^+^S100β^+^ astrocytes. Scale bars, 200 μm or 20 μm in magnification

To examine whether activation of HSF1 is efficient in reducing the number of A1 astrocytes, the rat following T8-T10 contusion received intraperitoneal injection of 100 μl of 20 mg/kg 17-AAG three times a week on alternate days. Control rats received DMSO alone. Western blot showed that protein levels of C3 in the injured cords were significantly decreased at 1 day and 4 days following agonist injection. Meanwhile, the reduction of HSP70 expression at lesion sites was markedly prevented by the treatment of 17-AAG (Fig. 8a–c). It was noteworthy that the agonist was inefficient in affecting the expression of C3 or HSP70 at 7 days due to the unknown reasons in vivo (Fig. 8a–c). Further immunostaining of C3-positive A1 astrocytes demonstrated that cell number of C3^+^/S100β^+^ was reduced by HSF1 agonist in comparison with those of the control at 1 day and 4 days following SCI (Fig. 9a, b). The data indicate that activation of HSF1 in the injured spinal cord is able to reduce the number of A1 astrocytes.

### Activation of HSF1 inhibits accumulation of reactive astrocytes at the lesion sites

**Fig. 10:**
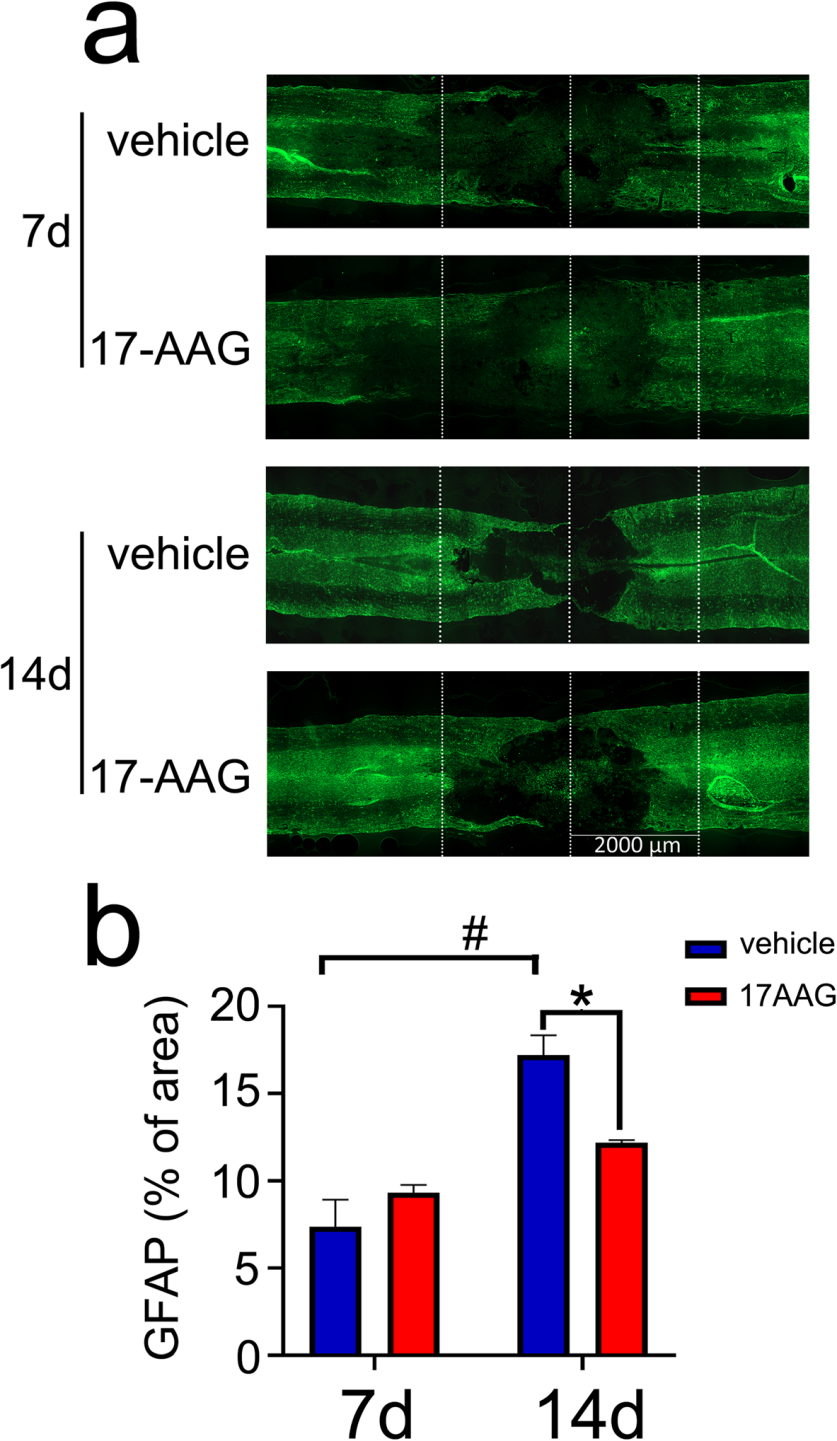
Immunostaining of GFAP-positive astrocytes at lesion sites of the injured cord following treatment with 20 mg/kg 17-AAG for 7 days and 14 days. **a** Area occupied by GFAP-positive scar-forming astrocytes within 2000 μm either side of the lesion center at 7 or 14 days after SCI. *n* = 6. **b** Statistical analysis of GFAP-positive percentage accounting for total area of 4000 μm border from six samples each 3 fields. Error bars represent the standard deviation, *P < 0.05, ^#^*P* < 0.05, two-way ANOVA with Tukey’s test. *F* (1, 2) = 32.20, *P* = 0.0297. 14 days vehicle vs 7 days vehicle, *P* = 0.0151; 14 days 17-AAG vs 14 days vehicle, *P* = 0.0255

Though functions of reactive astrocytes in CNS recovery are still controversial with either detrimental or supportive, but excessive astrogliosis will lead to harmful effects by exacerbating inflammation or inhibiting synapse sprouting and axon growth [40]. To observe effects of HSF1 activation on the accumulation of reactive astrocytes, especially the neurotoxic A1 phenotypes at the lesion sites, 17-AAG was administered to the rat following spinal cord contusion. Longitudinal sections stained with GFAP displayed that the number of reactive astrocytes at lesion sites was significantly reduced at 7 days and 14 days, as evaluated by the percentage of GFAP-positive area covering the lesion border with 2 mm rostral and caudal to the epicenter (Fig. 10a, b). The results indicate that activation of HSF1 is beneficial for decreasing the accumulation of reactive astrocytes at the lesion sites.

## Discussion

Molecular screening studies have revealed that A1 and A2 subtype of reactive astrocytes are detectably expressed in various CNS neurodegenerative disorders and injured spinal cord [4, 13, 41–46]. A1 astrocytes are assigned to be neurotoxic by upregulating many genes associated with the synapse and neuronal degeneration [4, 6]. As such, A1 astrocytes are known as disease-associated or dysfunction-associated cell types in the neurodegenerative disorders and the severed cord [4, 45]. In fact, block of A1 astrocyte conversion in the neuropathological CNS contributes to the functional recovery [4, 45]. HSF1-mediated suppression of A1 astrocytes in the injured cord might be beneficial for the alleviation of neuropathology. However, insights into reactive astrocyte states and their dynamic conversion in the microenvironment of the pathological tissue are still challenges, due to heterogeneity of reactive astrocytes and complex regulatory signaling. Based on molecular signatures identified by transcriptome profiling, C3 and S100A10 are respectively recognized as the feasible markers of A1 and A2 astrocytes, and were adopted to analyze relations of the two cell subtypes with the CNS pathology [4, 14, 44]. In the present study, we examined the distribution of A1 and A2 astrocytes using the according markers at lesion sites of the cord, and found that HSF1 was a potential target in blocking A1 astrocyte conversion following SCI.

The phenotype conversion of reactive astrocytes strongly depends on the injury types of CNS. For example, a systemic injection of LPS promotes transition of A1, whereas ischemia induces A2 astrocytes [10]. Once switched to the A1 astrocytes, they produce a variety of proinflammatory cytokines such as TNF-α, IL-1β, and complement component C3, as well as several C-X-C class of chemokines [7, 10]. Meanwhile, the expression of anti-inflammatory cytokines including IL-4 and IL-10 is significantly downregulated. Here, we displayed that injury-induced expression of HSF1 was dynamically regulated in the astrocytes following SCI. Many studies have shown that HSF1 protein is a key regulator of innate immunity [47]. It has been found to repress transcription of the IL-1β gene through physical interaction with the nuclear factor of interleukin 6 [27]. The protein can also inhibit transcription of the TNF-α gene by binding to the promoter [28]. In the model of mouse liver ischemia/reperfusion injury, HSF1 has been found to inhibit the activation of the NLRP3 inflammasome through activating β-catenin [26]. Therefore, HSF1 function in inhibiting the conversion of A1 astrocytes is possibly attributed to suppressing inflammatory activation of reactive astrocytes. It is interesting to note that interference of HSF1 expression in the astrocytes has also resulted in the reduction of C3 protein levels, suggesting the biological importance of HSF1 expression stability in the regulation of astrocyte phenotypes. In unstressed cells, the HSF1 monomer is retained in the cytoplasm in a complex with HSP40, HSP70, and HSP90, as well as the TRiC [20]. The decrease in HSF1 protein levels may lead to the increase of unbound chaperone proteins including HSP90, which in turn plays roles in suppressing the expression of C3 and inflammatory cytokines, as shown in ischemic postconditioning-induced cardioprotection [48]. However, the postulation needs to be further clarified.

Upon various stresses including heat shock, oxidants, and proteotoxic agents, HSF1 translocates to the nucleus in the form of a homotrimer that drives activation of many protein chaperones to protect cells from proteotoxicity and cell death [49]. Hsp70 is one of the canonical HSF1 target genes, which performs multiple pathophysiological functions such as mediating tumor progression, peripheral and central neuropathies, and innate immunity [50–53]. It has been shown that HSF1 induces expression of hsp70 to inhibit the production of proinflammatory cytokines in monocytes and macrophages under stimulation of moderate alcohol [54, 55]. In the present study, we showed that application of 17-AAG resulted in the activation of HSP70 in the astrocytes, suggesting that HSF1-mediated inhibition of C3 in reactive astrocytes might be involved in the action of HSP70.

C3 is inducibly expressed in astrocytes of most neurodegenerative diseases by inflammatory cytokines such as IL-1β, IFN-γ, and TNF-α [39, 56, 57]. Several protein kinases including MKK6, P38, and NFκB have been shown to affect C3 production [39, 58]. NFκB-activated astroglial release of C3 contributes to synaptic dysfunction in Alzheimer’s disease [58]. In the present study, we found that activation of HSF1 decreased the protein levels of C3 in astrocytes under the regulation of the MAPK/NFκB axis, suggesting that HSF1-mediated NFκB inactivation is involved in suppressing C3 expression. As an important transcription factor, HSF1, together with its inducible HSP70 protein, has been shown to exert anti-inflammatory effects through multiple signal pathways [59]. In addition to a direct role in inhibiting the expression of TNF-α and IL-1β through binding to the promoters, HSF1 is able to regulate the activity of NFκB through either reducing the degradation of IκB protein [60] or inhibiting the nuclear binding activity of NFκB [47]. Many inflammatory cytokines and reactive oxygen species (ROS) are robust activators of MAPK signaling [61]. As such, the indirect effect of HSF1 in inhibiting the activities of MAPKs is possibly attributed to the negative regulation of several inflammatory cytokines or ROS, which in turn decrease the activation of MAPKs as evidenced in cardiac myocytes [25]. However, the relevant mechanisms remain to be elucidated.

## Conclusions

Protein levels of HSF1 were dynamically regulated in the reactive astrocytes following SCI, which was involved in inhibiting the A1 astrocyte conversion at lesion sites. HSF1-mediated regulation of astrocyte phenotypes was attributed to suppressing the activity of NFκB, which in turn repressed the expression of complement component C3.

## Supplementary Information

**Additional file 1: Figure S1.** Western blot analysis of HSF1 expression following spinal cord contusion at 0d, 1d, 4d and 7d, respectively. Quantities were normalized to endogenous β-actin. n = 6. Experiments were performed in triplicates. Error bars represent the standard deviation (P > 0.05).

**Additional file 2: Figure S2.** Immunostaining of HSF1 in the cross sections of rat contused spinal cord showed colocalization with S100β-positive cells at 0d, 1d, 4d and 7d, respectively. Rectangle indicates region magnified. Arrowheads indicate colocalization of HSF1 with astrocytes. Scale bars, 200 μm or 50 μm in magnification.

**Additional file 3: Figure S3.** Determination of C3 ptotein levels in the astrocytes following inhibition of HSF1 expression. **a** Western blot analysis of HSF1 and C3 following astrocyte transfection with HSF1 siRNA for 48 h. **b** Quantification of (**a**). **c** Western blot analysis of HSF1 and C3 following astrocyte treatment with 0 - 60 μM quercetin for 24 h. **d** Quantification of (**c**). Quantities were normalized to endogenous β-actin. n = 6. Experiments were performed in triplicates. Error bars represent the standard deviation. *P < 0.05, one-way ANOVA with Dunnett’s post hoc test.

## Data Availability

The datasets used and/or analyzed during the current study are available from the corresponding author on reasonable request.

## Abbreviations

ANOVA: Analysis of variance
HSF1: Heat shock transcription factor 1
HSP70: Heat shock protein 70
AD: Alzheimer’s disease
MAPK: Mitogen-activated protein kinase
NFκB: Nuclear factor kappa-B
C3: Complement component 3
17-AAG: 17-Allylamino-17-demethoxygeldanamycin
DMSO: Dimethyl sulfoxide
SDS: Sodium dodecyl sulfate
SCI: Spinal cord injury
GFAP: Glial fibrillary acid protein
