# Supplementary material for: HSF1 is involved in suppressing A1 phenotype conversion of astrocytes following spinal cord injury in rats
Source: J Neuroinflammation. 2021 Sep 16;18:205. doi: 10.1186/s12974-021-02271-3 (PMC8444373; doi:10.1186/s12974-021-02271-3)
Supplement: Supplementary file 1 — Additional file 1: Figure S1. Western blot analysis of HSF1 expression following spinal cord contusion at 0d, 1d, 4d and 7d, respectively. Quantities were normalized to endogenous β-actin. n = 6. Experiments were performed in triplicates. Error bars represent the standard deviation (P > 0.05). [file 12974_2021_2271_MOESM1_ESM.docx]

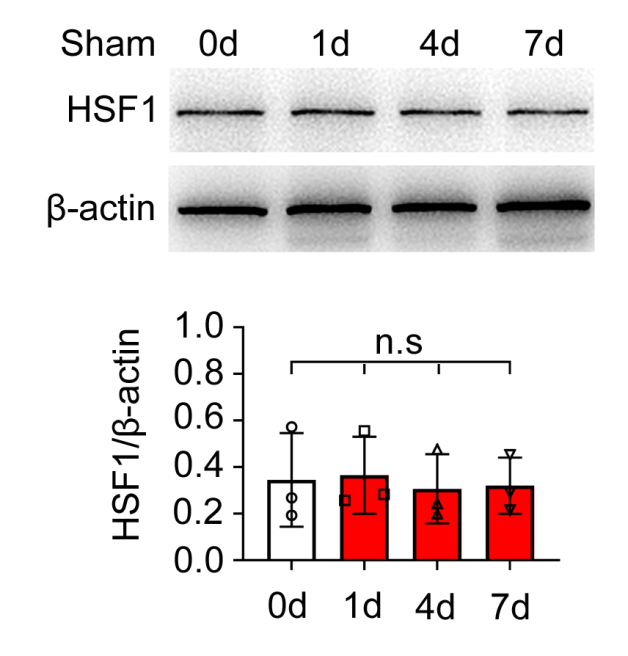


Figure S1. Western blot analysis of HSF1 expression following spinal cord contusion at 0d, 1d, 4d and 7d, respectively. Quantities were normalized to endogenous β-actin. n = 6. Experiments were performed in triplicates. Error bars represent the standard deviation (P > 0.05).
