# Supplementary material for: HSF1 is involved in suppressing A1 phenotype conversion of astrocytes following spinal cord injury in rats
Source: J Neuroinflammation. 2021 Sep 16;18:205. doi: 10.1186/s12974-021-02271-3 (PMC8444373; doi:10.1186/s12974-021-02271-3)
Supplement: Supplementary file 2 — Additional file 2: Figure S2. Immunostaining of HSF1 in the cross sections of rat contused spinal cord showed colocalization with S100β-positive cells at 0d, 1d, 4d and 7d, respectively. Rectangle indicates region magnified. Arrowheads indicate colocalization of HSF1 with astrocytes. Scale bars, 200 μm or 50 μm in magnification. [file 12974_2021_2271_MOESM2_ESM.docx]

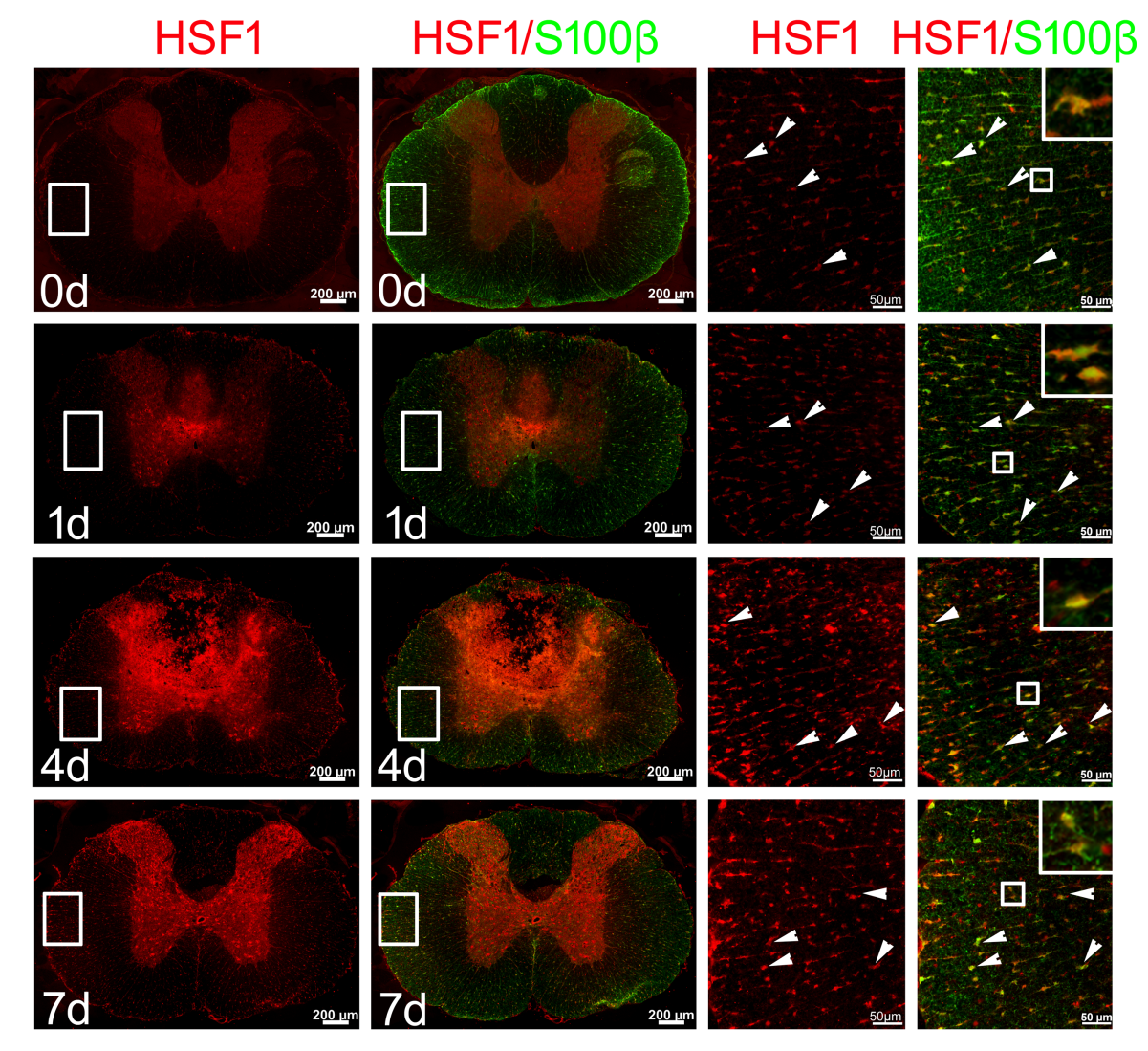


Figure S2. Immunostaining of HSF1 in the cross sections of rat contused spinal cord showed colocalization with S100β-positive cells at 0d, 1d, 4d and 7d, respectively. Rectangle indicates region magnified. Arrowheads indicate colocalization of HSF1 with astrocytes. Scale bars, 200 μm or 50 μm in magnification.
