# Supplementary material for: HSF1 is involved in suppressing A1 phenotype conversion of astrocytes following spinal cord injury in rats
Source: J Neuroinflammation. 2021 Sep 16;18:205. doi: 10.1186/s12974-021-02271-3 (PMC8444373; doi:10.1186/s12974-021-02271-3)
Supplement: Supplementary file 3 — Additional file 3: Figure S3. Determination of C3 ptotein levels in the astrocytes following inhibition of HSF1 expression. a Western blot analysis of HSF1 and C3 following astrocyte transfection with HSF1 siRNA for 48 h. b Quantification of (a). c Western blot analysis of HSF1 and C3 following astrocyte treatment with 0 - 60 μM quercetin for 24 h. d Quantification of (c). Quantities were normalized to endogenous β-actin. n = 6. Experiments were performed in triplicates. Error bars represent the standard deviation. *P < 0.05, one-way ANOVA with Dunnett’s post hoc test. [file 12974_2021_2271_MOESM3_ESM.docx]

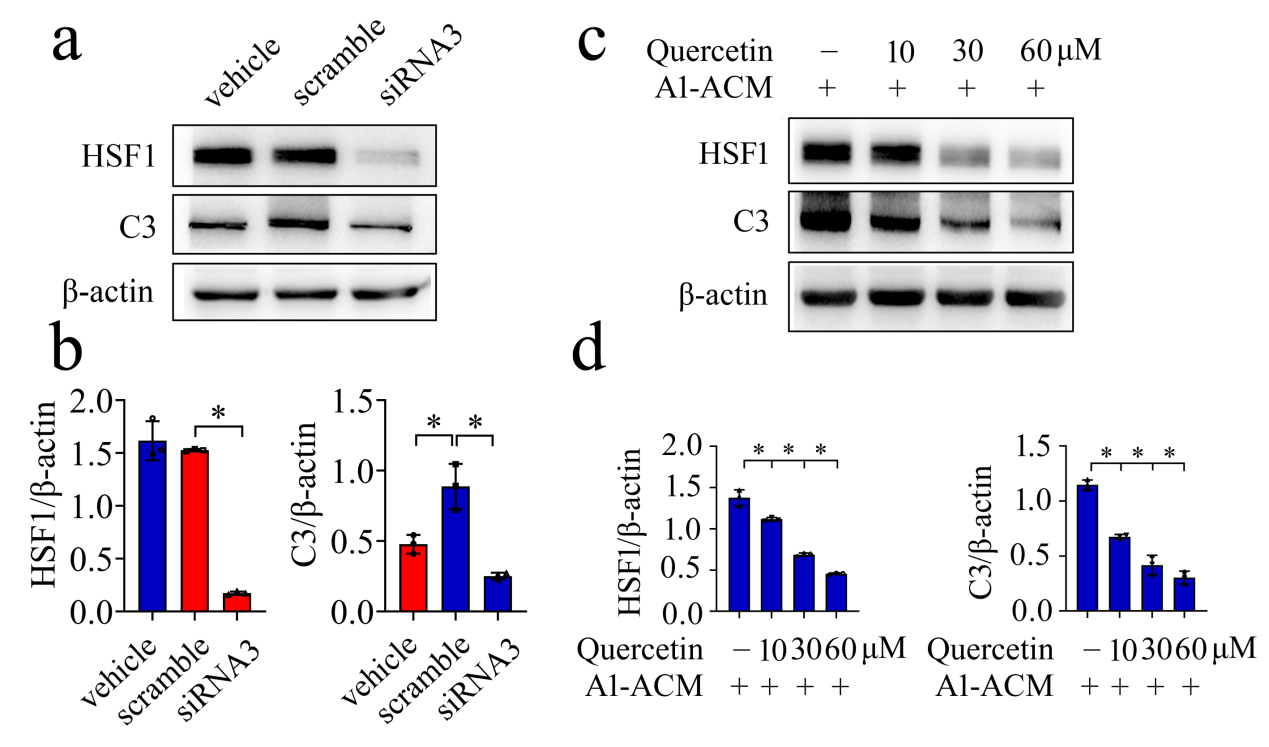


Figure S3. Determination of C3 ptotein levels in the astrocytes following inhibition of HSF1 expression. **a** Western blot analysis of HSF1 and C3 following astrocyte transfection with HSF1 siRNA for 48 h. **b** Quantification of (**a**). **c** Western blot analysis of HSF1 and C3 following astrocyte treatment with 0 - 60 μM quercetin for 24 h. **d** Quantification of (**c**). Quantities were normalized to endogenous β-actin. n = 6. Experiments were performed in triplicates. Error bars represent the standard deviation. **P* < 0.05, one-way ANOVA with Dunnett’s post hoc test.
